# Promotion of couples’ voluntary HIV counseling and testing in Lusaka, Zambia by influence network leaders and agents

**DOI:** 10.1186/1742-4690-9-S2-P210

**Published:** 2012-09-13

**Authors:** K Wall, W Kilembe, A Nizam, C Vwalika, M Kautzman, E Chomba, A Tichacek, G Sardar, D Casanova, F Henderson, J Mulenga, D Kleinbaum, S Allen

**Affiliations:** 1 Emory University, Atlanta, GA, USA; 2 Rwanda Zambia HIV Research Group, Atlanta, GA, USA

## Background

In sub-Saharan Africa, most HIV transmissions occur in stable heterosexual relationships. Couples' voluntary HIV counseling and testing [CVCT] is an effective strategy targeting this at-risk group. This study identified predictors of successful CVCT promotion in Lusaka, Zambia.

## Methods

CVCT promotions were conducted by influential network leaders [INLs] who identified agents [INAs], who in turn delivered CVCT invitations from over an 18-month period, with a mobile unit crossing over from one intervention neighborhood to another at 9 months. INA, couple, and invitation characteristics predictive of couples' testing were evaluated accounting for two-level clustering.

## Results

320 INAs delivered 29,119 invitations resulting in 1727 couples testing (6% success rate). In multivariate analyses, INA characteristics significantly predictive of CVCT uptake included promoting in community-based (adjusted odds ratio [aOR]=1.3) or health (aOR=1.5) networks versus private networks, being employed in the sales/service industry (aOR=1.5) versus unskilled manual labor, owning a home (aOR=0.7) versus not, and testing for HIV with a partner (aOR=1.4) or alone (aOR=1.3) versus never testing. Cohabiting couples were more likely to test (aOR=1.4) than non-cohabiting couples. Context characteristics predictive of CVCT uptake included inviting couples (aOR=1.2) versus individuals, the woman (aOR=1.6) or couple (aOR=1.4) initiating contact versus the INA, the couple being socially acquainted with the INA (aOR=1.6) versus having just met, home invitation delivery (aOR=1.3) versus elsewhere, and easy invitation delivery (aOR=1.8) versus difficult as reported by the INA. Use of mobile units was very low and did not substantially contribute to CVCT service delivery.

## Conclusion

This study demonstrated the ability of influential people to promote CVCT and identified agent, couple, and context-level predictors of CVCT uptake in Lusaka, Zambia. We encourage the development of CVCT promotions in other sub-Saharan African countries to support sustained CVCT dissemination.

